# Perceiving the Self and Emotions with an Anxious Mind: Evidence from an Implicit Perceptual Task

**DOI:** 10.3390/ijerph182212096

**Published:** 2021-11-18

**Authors:** Michella Feldborg, Naomi A. Lee, Kalai Hung, Kaiping Peng, Jie Sui

**Affiliations:** 1 School of Psychology, University of Aberdeen, Aberdeen AB24 3FX, UK; michella.feldborg@abdn.ac.uk (M.F.); n.lee.20@abdn.ac.uk (N.A.L.); jie.sui@abdn.ac.uk (J.S.); 2 Department of Psychology, Tsinghua University, Beijing 100084, China; pengkp@mail.tsinghua.edu.cn

**Keywords:** anxiety, mental health, self-prioritisation effect, positivity bias

## Abstract

Anxiety disorders cause mental distress and low wellbeing in many people worldwide. Theories of anxiety describe negative worldviews and self-views as maintaining factors of the disorders. Recent research in social cognition has found a link between depression and altered perceptual biases to emotions, but the same research on anxiety is still missing. In this study, we measured perceptual biases to emotional and self-related stimuli in sub-clinically anxious participants and healthy controls using a self-emotional shape-label matching task. Results demonstrate that anxious participants had a diminished perceptual self-bias compared with healthy controls. Furthermore, the severity of anxiety was related to an emotional bias towards valanced other-related stimuli. The findings confirm the hypothesis that anxious individuals display an altered self-prioritisation effect in comparison with healthy individuals and that anxiety severity is linked to altered responses to emotionally valanced others. These findings have potential implications for early diagnosis and treatment of anxiety disorders.

## 1. Introduction

Anxiety disorders are some of the most common mental health issues. Between 13% and 33.7% of people in the Western world experience anxiety at some point in their lifetime [1]. Anxiety disorders are characterised by persistent, excessive, and debilitating fear and worry over time, but the manifestation of anxiety can vary from person to person. Common physical symptoms are trembling, increased heartrate, nausea, restlessness, and headaches. Psychological symptoms include feeling tense and nervous, a sense of doom, uncontrollable worrying, and ruminating. The main behavioural symptom is avoiding anxiety-triggering situations [2]. Anxiety can have detrimental consequences, including issues with maintaining relationships, meeting new people, keeping a job, and enjoying life. Treatments of anxiety disorders include various forms of therapies and medications which successfully alleviate symptoms in around 6 out of 10 people [3].

Current research on mental health has found links between mood disorders and perceptual processes [4,5,6,7], but research into anxiety and self-perception is still lacking. Anxiety is theorised to be linked to an altered view of oneself and the world [8,9,10,11,12], where the anxious individual focusses excessively on threats and dangers. The theory applies to various disorders such as social anxiety and phobias, but the present study focused on generalised anxiety disorder, which is often measured due to the overlap with other anxiety and depression disorders [3]. The aim of the current study was to understand the psychological mechanisms of anxiety in the non-clinical population by using cognitive tasks to measure perception of the self and emotions. Here, we specifically focussed on two perceptual biases: the self-prioritisation effect and the positivity bias.

The self-prioritisation effect, a strong bias towards self-related stimuli, is a robust finding in cognitive psychology [13,14,15]. Dichotic hearing tasks are the classic way to test the self-prioritisation effect [13,16]. In the task, participants focus on the input in one ear, while ignoring the input from the other ear. The input most frequently noticed in the ignored ear is the mentioning of the participant’s own name. Memory tests can also measure a self-prioritisation effect; for example, people remember more items from their “own shopping basket” than from a stranger’s [17]. The mechanisms underpinning self-prioritisation are still controversial, and noteworthy attempts to explain the effect have relied on everything from attention over memory to perception [4,6,7]. In the current study, we investigated the self-prioritisation effect in implicit, visual perception.

An experimental paradigm has recently been developed in cognitive psychology and neuroscience to capture differences in various aspects of the self [18,19,20]. In the shape-label perceptual matching paradigm, participants are shown a shape (e.g., circle) and are told that this specific shape represents themselves, while other shapes represent other people (e.g., a friend or a stranger). The task is to judge whether the shape is presented with the matching label as quickly and accurately as possible. People are quicker and more accurate to identify whether a shape-label pair matches when the pairing represents themselves. Even though the task does not require conscious self-perception, the self-prioritisation effect is still present. This shows that most people have an implicit perceptual bias that makes them prioritise processing of self-relevant information in the environment around them. In addition, the positivity bias, a preference towards positive stimuli, is also common in healthy people. People naturally pay more attention to emotional stimuli, especially when positive and relevant to self-preservation [21,22,23], but also when the stimuli are negative and arousing [4]. People tend to remember positive memories more frequently and better than neutral or negative memories. The positivity bias can also be measured using the shape-label matching paradigm. Stolte et al. (2017) [24] and McIvor et al. (2020) [4] adjusted the perceptual matching task so that each shape was presented with an emotional expression. The healthy control group in both studies replied faster and more accurately on trials with positive expressions than they did to neutral or negative expressions. There was also improved performance on trials with negative expressions compared with the neutral ones. People usually focus more on emotional stimuli, especially when the stimuli are positive.

The underlying mechanisms of the self-prioritisation effect and the positivity bias are still debated. One possibility is that the two biases are underpinned by the same neural substrate, the medial prefrontal cortex [25]. A combined self-positivity bias can be tested using a shape-label matching task which distinguishes between “good” and “bad” in addition to “self” and “other” [26]. Healthy people respond most efficiently to the shape that represents their good self. Similarly, the self-prioritisation effect is boosted in a positive-self connection and dampened in a negative-self connection [22], suggesting that humans generally seek a positive view on themselves and the world. However, Schäfer and Frings (2019) [27] were unable to find a link between self-esteem and self-prioritisation, showing that the connection between positivity and self is not detangled just yet.

People with mental disorders might categorically divert from the common perceptual biases towards positivity and the self. As an example, depression weakens the positivity and negativity biases [4]. In a shape-label matching task paired with happy, neutral, and sad facial expressions, healthy controls showed strong positivity and self-biases and a small negativity bias. Depressed participants had a normal self-bias but a reduced bias towards positive or negative stimuli, leaving them insensitive to emotional biases. This indicates that depression is characterised by an indifference to emotional input, which might be causing, maintaining, or worsening the disorder. Depression and anxiety are co-morbid and often occur together. The present study explored whether anxiety is also linked to divergent perceptual biases.

Previous research has found links between explicit negativity bias and anxiety. Kalenzaga and Jouhaud (2018) [28] found that socially anxious participants remembered as many self-relevant and positive words as controls. However, when the participants were asked to “remember” instead of “guess” words on the list, the positivity bias disappeared in anxious participants. Adding a need for certainty meant that the anxious participants had reduced positivity bias. Muranaka and Sasaki (2018) [6] tested word generation in social anxiety. Participants had to come up with words to describe a negative and embarrassing social situation. The socially anxious participants engaged in more negative and dysfunctional self-rumination than controls. They generated more negative words overall and even more negative self-related words than non-anxious people. The converging evidence indicates that anxiety (specifically social anxiety) can reduce the positivity bias and the self-prioritisation effect in memory. However, whether the reduction extends to implicit biases is yet to be explored.

To investigate how anxiety, the self-prioritisation effect, and the positivity bias are related in perceptual processes, we must draw from theories on cognition and perception in anxiety disorders. Cognitive and attentional theories describe a threat-related bias which gives anxious people a larger attentional bias towards threats compared with non-anxious people [29,30]. The theory is supported by the Information-Processing Model of Anxiety [9,10], according to which anxious individuals have heightened threat detection, meaning that they are more likely to perceive threats even when there are none. As a result, they are more likely to activate an automatic threat response and later to consciously reflect and ruminate on the threatening situation. The theory has a foundation in findings from attention tasks which show that anxious people engage more with and reply quicker to threatening input and situations [31,32,33,34], as well as evidence of threatening stimuli distracting anxious people from performing well [35]. Given the heightened focus on threats, anxious individuals might show reduced positivity bias.

Anxiety is also characterised by an altered self-view [8,11,12], which often takes the form of enhanced negative self-focus. Anxious individuals ruminate on their past and future experiences. Since this rumination is dysfunctional and focusses excessively on negative experiences, anxious people often have distorted evaluations of themselves which in turn can lead to decreased self-esteem. Anxious people are more likely to remember self-threatening information which challenges a positive self-view, whereas healthy people forget self-threatening information to preserve their self-image [31]. The changes in how anxious individuals view themselves could be linked to a divergent self-prioritisation effect.

On the technical level, it is vital to test changes in biases using both explicit and implicit tasks because anxiety is a disorder in which these processes may differ. Here, we focus on the implicit biases, using the self-emotional shape-label matching task that allows manipulating personal associations and emotions on the implicit level. Thus, the current study measured both the self-prioritisation effect, the positivity bias, and the intercept between the two under the same metric. It is also important to make a distinction between (1) the differences in perceptual biases between anxious and non-anxious individuals and (2) the relationship between perceptual biases and anxiety severity within the anxious population. For this reason, we investigated both the group differences and the trends on the individual level. Lastly, we measured generalised anxiety disorder to investigate how symptoms of worrying and ruminating are linked with self- and emotional biases, unlike previous memory studies [6,28] that focused on social anxiety and the perceived emotions of others.

Overall, from the previous studies, e.g., [4,18,29,30,31], we predicted that:

1. Self-positivity would be diminished in the anxious group. If this were true, it would be reflected in slower and less accurate responses to self-related and positive stimuli in the anxious group;
2. Self-positivity would be related to the severity of anxiety, although the nature of this relation could not be predicted based on the current knowledge. This hypothesis would be supported if faster or slower responses to self-related or emotional stimuli could predict anxiety levels.

## 2. Materials and Methods

### 2.1. Participants

No a priori power analyses were performed to determine sample size given that this experiment was conducted as a pilot study. For clinical trials, a sample size between 25 and 50 is considered reasonable [36]. The sample size was set to 42 in each group to allow for full stimuli counterbalancing. Eighty-four undergraduate students from a British university participated in the experiment in exchange for course credits. A total of 13 participants identified as men, 70 as women, and one as a different gender, with the mean age of 21.24 years old (SD = 4.09, range from 18–51). Half the participants were randomly assigned to the shape-label matching task with “self” and “friend” labels and the other half with “self” and “stranger” labels. Participants were recruited through the online recruitment system of the psychology department. The study was approved by the School of Psychology’s Ethics Committee (protocol code PEC/4535/2020/8 and date of approval 6 October 2020).

### 2.2. Stimuli

**Figure 1:**
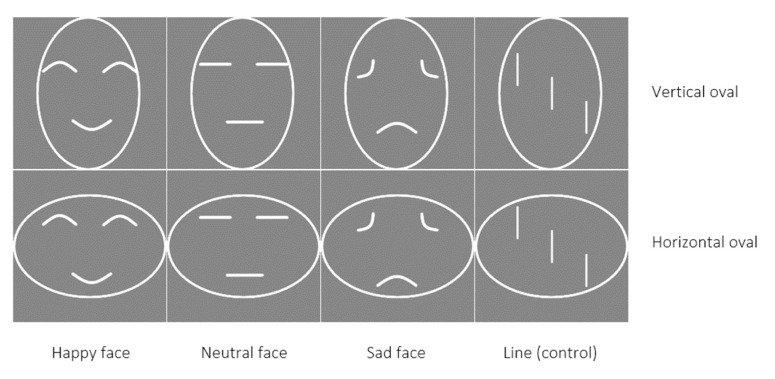
Emotional stimuli used in the shape-label matching task.

A vertical and horizontal outline of an oval were randomly associated with the self and friend or stranger. The shape contained three lines that formed a happy face, a neutral face, a sad face, or three vertical lines (see Figure 1). A white fixation cross was displayed in the centre of the screen (50%, 50%). Shapes were presented above the fixation cross (50%, 35%) and white labels were presented below the fixation cross (50%, 65%). All stimuli were shown against a grey background. The experiment was conducted online through Inquisit [37].

### 2.3. Procedure and Measures

#### 2.3.1. Self-Emotional Shape-Label Matching Task

The self-biases were measured using the perceptual self-emotional shape-label matching task. The participant was told that each shape (vertical oval or horizontal oval) represented a person—themselves and their friend or a stranger. To make the friend and stranger as concrete as the self, the participant named them prior to the experiment. The task was to judge if the presented shape-label pairing corresponded with the previously learned association. The participants were told that the lines were irrelevant to the task.

**Figure 2:**
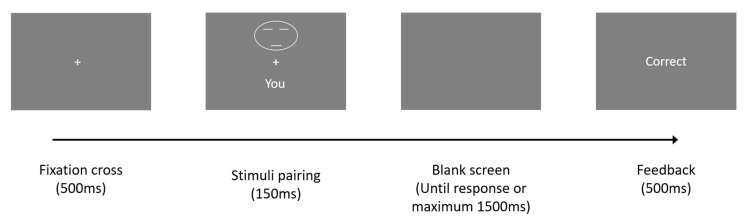
Flow chart of the perceptual shape-label matching task.

Each trial had four stages (see Figure 2). First, a blank screen with a fixation point was present for 500 ms. Next, the stimuli were shown for 150 ms and consisted of a shape above the fixation point and a word below it: “You”, “Friend”, and “Stranger”. A blank screen was then shown while the participant indicated whether the shape and word matched one of the learned associations. This screen was present until the participant answered or the reaction time surpassed 1500 ms. The last screen provided feedback by stating “Correct”, “Incorrect”, or “Too slow” for 500 ms.

There were three short practice blocks (eight demo trials, eight self-paced practice trials, and six real-time practice trials). In the demo trials, the shape-label pair remained on the screen with instructions until a response was made. The instruction indicated whether the pair was a match or not and the response key needed (i.e., “MATCH! So, you’d press ‘v’ with the index finger of your left hand”; or “NOT A MATCH! So, you’d press ‘b’ with the index finger of your right hand. Remember this shape matched with…”.). In the self-paced practice trials the shape-label pair remained on screen until a response was made, but no instruction was provided. The real-time practice trials were identical to the experimental trials.

The formal experiment contained four blocks. Each of the four formal blocks contained 80 trials, giving 320 experimental trials in total. There were 24 distinct combinations of conditions (Person: self, friend or stranger; Emotion: happy, line, neutral, sad; Matching: match, mismatch). Each participant was exposed to 16 conditions, as one half completed the friend conditions, while the other completed the stranger conditions (see Table 1). The entire shape-label matching task including practice trials took approximately 15 min to complete. This version of the task was shorter and easier than the original lab-based shape-label matching task to accommodate for participants doing it online using their own computer [38].

#### 2.3.2. Anxiety

**Figure 3:**
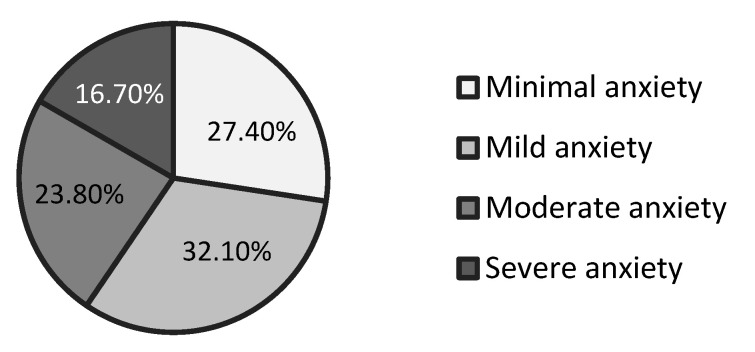
Distribution of participants on the General Anxiety Disorder questionnaire in percentages.

Participants’ anxiety levels were measured with the Generalised Anxiety Disorder questionnaire (GAD-7) [39]. GAD-7 consists of 7 symptoms that measure generalised anxiety in the past two weeks. Participants were asked to rate how often they had experienced each symptom over the last two weeks, which gives a long-term picture of their anxiety severity. Symptoms were rated from 0 (not at all) to 3 (nearly every day). The sum of all 7 answers placed participants in one of the following categories: minimal anxiety (0–4), mild anxiety (5–9), moderate anxiety (10–14), and severe anxiety (15–21). Participants were evenly spread across the GAD-7 spectrum (see Figure 3). We used continuous scores in the regression analyses and a division at 5 in the analyses of variance (ANOVA) (<5 = non-anxious group, ≥5 = anxious group). The cut-off score at 5 ensured a reliable distinction between healthy individuals and people with subclinical anxiety [40,41]; the latter suggests cut-off at 6. The internal reliability of GAD-7 in this study was Cronbach’s α = 0.894. As GAD is highly co-morbid not only with other anxiety disorders but also with depression disorders, depressive symptoms were measured as a control factor using the Beck Depression Inventory, BDI-II (see footnote in Table 2).

### 2.4. Data Analysis

All 84 participants were included in the group-level analysis. The respective responses to “friend” and “stranger” were merged into the same variable named “other” since there were neither main effects of the self-friend and self-stranger biases nor interactions with emotional biases (see Appendix A, Table A1). By merging the two groups into one, the statistical tests gained more power, and the number of violated statistical assumptions was lowered, while the analysis could still detect self-biases. The emotional conditions were compared with the control condition, showing that the three vertical lines and sad expressions elicited very similar performance, while the neutral and happy stimuli facilitated better performance (see Appendix A, Table A2).

Implicit self-bias was measured by comparing self-related trials with other-related (friend/stranger) trials on reaction time and response accuracy. We measured biases towards emotional stimuli by comparing response time and accuracy with the happy, line, neutral, and sad stimuli. In addition, we used the emotion responses to measure a combination of self- and emotion-bias e.g., the self-positivity bias.

#### 2.4.1. Are the Self-Bias and Positivity Bias Weakened in the Anxious Group Compared with Controls?

ANOVAs were run to explore the groupwise effects of anxiety on self-biases. We conducted repeated-measures ANOVA (rmANOVA) on response accuracy and reaction time with the two within-subjects variables, emotion (Happy × Line × Neutral × Sad) and person (Self × Other), and with anxiety (non-anxious vs. anxious) as the between-subjects variable.

#### 2.4.2. What Are the Individual Differences in Self-Bias, Positivity Bias, and Severity of Anxiety?

The regression analysis was used to better understand the relationship between reaction times and anxiety in anxious individuals. We applied a hierarchical regression analysis between anxiety score and reaction times in the anxiety group (GAD ≥ 5). We used the reaction times in the regression analysis because they were similar to but more sensitive than the accuracy scores. The reaction times to sad, neutral, and happy expressions were used to create three models predicting the severity of anxiety. The first step accounted for age and depression, the second step accounted for age, depression, and response times to self-related stimuli, and the third step accounted for age, depression, and the response times to both self- and other-related stimuli (an overview of the steps is presented in Table 3 in the Section 3).

## 3. Results

### 3.1. Are the Self-Bias and Positivity Bias Weakened in the Anxious Group Compared with Controls?

The ANOVAs on accuracy showed strong support for the main effect of person (self vs. other), F(1,82) = 15.01, *p* < 0.001, η^2^ = 0.053, *BF*_10_ = 6.83 × 10^11^. More accurate responses were made to the self-related stimuli (0.84 ± 0.13) than other-related stimuli (0.76 ± 0.15). No evidence was found for the main effect of emotion (F(3,246) = 0.39, *p* = 0.76, η^2^ = 0, *BF*_10_ = 0.01) or for the main effect of anxiety, F(1,82) = 0.78, *p* = 0.38, η^2^ = 0.004, *BF*_10_ = 0.30. Additionally, no significant effect of interactions were observed, either for Person × Anxiety (F(1,82) = 0.47, *p* = 0.493, η^2^ = 0.002, *BF*_10_ = 0.17), Emotion × Anxiety (F(3,246) = 0.17, *p* = 0.915, η^2^ = 0, *BF*_10_ = 1.13 × 10^−4^), Emotion × Person (F(3,246) = 0.46, *p* = 0.712, η^2^ = 0.001, *BF*_10_ = 3.87 × 10^−4^), or Emotion × Person × Anxiety (F(3,246) = 0.24, *p* = 0.869, η^2^ = 0, *BF*_10_ = 1.79 × 10^−7^). Participants performed more accurately on self-related trials, but there were no other significant main effects or interaction effects of accuracy.

The ANOVA on reaction times revealed strong evidence for the main effect of person, F(1,82) = 24.52, *p* < 0.001, η^2^ = 0.05, *BF*_10_ = 1.92 × 10^10^. There were faster response times to self-related stimuli (699 ± 83.3) than to other-related stimuli (738 ± 99.2). No evidence for the main effect of emotion, F(3,246) = 3.68, *p* = 0.013, η^2^ = 0.004, *BF*_10_ = 0.24, or for the group effect of anxiety was found, F(1,82) = 0.08, *p* = 0.776, η^2^ = 0.001, *BF*_10_ = 0.31.

**Figure 4:**
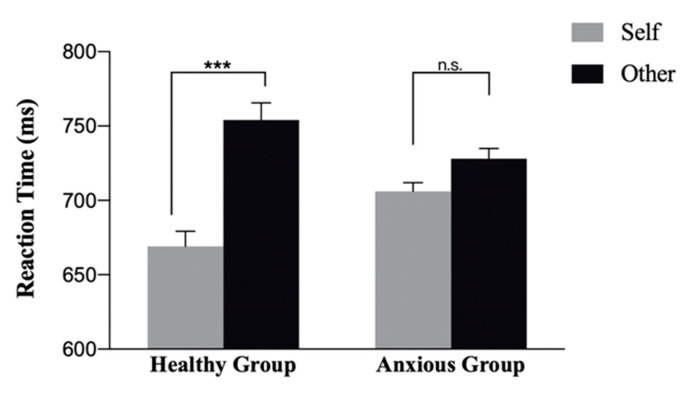
Results of the self-prioritisation effect in reaction time (ms). Error bars present standard error of means. n.s.: not significant, ***: *p*-value < 0.001.

The evidence for the interaction between person and anxiety group was strong, F(1,82) = 8.45, *p* = 0.005, η^2^ = 0.018, *BF*_10_ = 81846. Follow-up analysis showed strong evidence that the reaction times to self-related stimuli were faster than to other-related stimuli in the non-anxious group, (mean ± std: 669.48 ± 98.8 vs. 754 ± 110, t(82) = −4.04, *p* < 0.001, *Cohen’s d_z_* = 0.81, 95% CI (0.51, 1.11), *BF*_10_ = 89371), while the evidence of the effect in the anxious group was inconclusive, (mean ± std: 706 ± 92.9 vs. 728 ± 108, t(82) = 1.95, *p* = 0.214, *Cohen’s d_z_* = 0.22, 95% CI (0.04, 0.40), *BF*_10_ = 1.66) (See Figure 4). In sum, participants scoring low on the anxiety scale responded quicker to self-related stimuli than other-related stimuli, but this effect was not found in participants scoring ≥ 5 on the GAD-7.

There were no interactions in reaction times between Emotion × Anxiety (F(3,246) = 0.57, *p* = 0.632, η^2^ = 0.001, *BF*_10_ = 0.01), Emotion × Person (F(3,246) = 1.84, *p* = 0.141, η^2^ = 0.002, *BF*_10_ = 0.01), or Emotion × Person × Anxiety (F(3,246) = 0.10, *p* = 0.394, η^2^ = 0.001, *BF*_10_ = 2.21 × 10^−4^).

### 3.2. What Are the Individual Differences in Self-Bias, Positivity Bias, and Severity of Anxiety?

**Figure 5:**
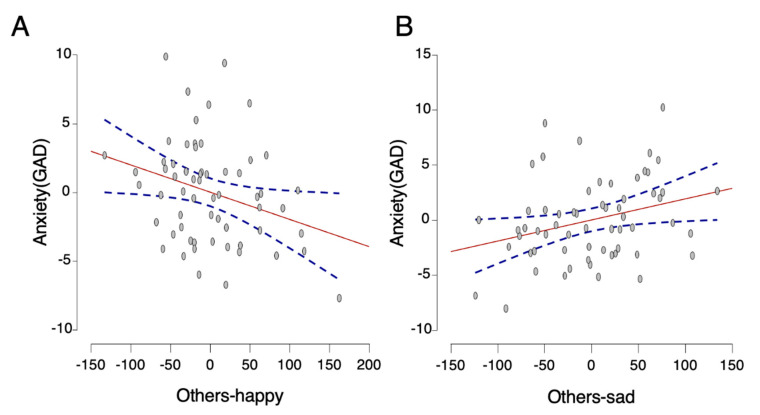
Relationship between severity of anxiety and reaction time to others’ expressions (happy and sad) in the anxious group. The partial regression plots illustrate the effect to valence of others’ facial expression by removing the effect of all other predictors. The associations are shown between GAD score with (**A**) reaction time to happy other-related stimuli and (**B**) reaction time to sad other-related stimuli. Note: The y-axis shows the residuals from regressing GAD scores and the x-axis shows the residuals from regressing reaction times (ms) on others-happy and others-sad. The linear fits are solid lines, and the 95% confidence interval are dashed lines. The GAD score was negatively associated with the reaction time to other-happy stimuli and positively associated with the reaction time to other-sad stimuli in anxious group.

Table 2 displays the means, standard deviations, and correlations for all study variables in the anxious group. As Table 3 shows, depression is a strong predictor of anxiety, F(2,57) = 8.38, *p* < 0.001, *R*^2^ = 0.23. Reaction times to all self-related stimuli failed to predict anxiety in step 2 (F(5,54) = 3.62, *p* = 0.007, *R*^2^ = 0.25). Importantly, after adding all predictors including control factors, step 3 was significant, F(8,51) = 3.56, *p* = 0.002, *R*^2^ = 0.36. Reaction times to other-happy stimuli was negatively correlated with anxiety (*β* = −0.48, *p* = 0.03). Reaction times to other-sad stimuli was a positive predictor of anxiety (*β* = 0.51, *p* = 0.029). In sum, the results show that in anxious individuals, faster responses to happy other-related stimuli and slower responses to sad other-related stimuli predicted more severe anxiety (see Figure 5).

## 4. Discussion

The current study aimed to examine implicit perceptual self- and positivity biases in sub-clinical anxiety and healthy controls. The results strongly support that the self-prioritisation effect is weakened in people with mild-to-severe anxiety compared with healthy controls. Within the anxious group, quicker responses to others with happy expressions and slower responses to others with sad expressions predicted increased severity of anxiety. The data indicate that the self-prioritisation effect was altered in anxious individuals in comparison with healthy controls and that the positivity bias was altered with increasing severity of anxiety.

We predicted that the sub-clinically anxious group would display a weakened self-prioritisation effect and positivity bias compared with healthy people based on current knowledge of similar self-prioritising processes in memory and attention [3,6,28]. The results show that the anxious group did not have a significant self-prioritisation effect, while the healthy group did, supporting the prediction and previous literature [4,8,11,12]. However, the group-level results did not find any links between anxiety and an altered positivity bias. In tasks that measure conscious cognition (e.g., memory tasks), anxiety is generally linked to a decrease in self-positivity bias, meaning that anxious people remember more negative self-related things and forget positive ones [6,28,31]. The current task measured implicit perceptual processing of the self and emotions. The self-prioritisation effect likely arose from implicit low-level information processes [15], but emotional biases might be more conscious or connected to different processes, which could explain why only a decrease in self-prioritisation was linked with experiencing anxiety.

The severity of anxiety, on the other hand, connected to an increase in the perceptual positivity bias. This answered our second question of how self-positivity was related to anxiety severity in the individual. Severe anxiety was related to improved performance on implicit happy stimuli, and reduced performance on sad stimuli. The quick responses to positive other-related input in highly anxious people can be explained using the Information-Processing Model of Anxiety [9,10]. Meta-analyses by Bar-Haim et al. (2007) [29] and more recently Günther et al. (2021) [30] concluded that anxious individuals have a small but robust early attentional bias towards threat. Although no threatening stimuli were present in this study, we found that emotional other-related faces were perceived differently by people with high anxiety. Happy faces were processed quicker, while sad faces were processed slower. It could be argued that happy faces were more arousing than sad faces, and that these results mirrored that alerted people reply quicker to arousing stimuli [23]. In this regard, it would be interesting to see how quickly anxious people perceive and process angry or surprised faces, which are more arousing and indicative of threats than the happy and sad faces used in this experiment. Alternatively, when an anxious individual implicitly processes negative stimuli slower, it might be because sad other-related expressions distract them from performing on the matching task. This interpretation would align with the interference theory in attention research [35,43]. However, since the non-emotional control stimuli and the sad expressions were highly comparable (see Appendix A, Table A2), it seems more likely that emotional stimuli facilitated, rather than distracted from, performance. In this light, severe anxiety was linked to happy faces improving performance more than in mild anxiety and to sad faces not improving performance as much as in mild anxiety. Either interpretation supports that perceptual and attentional biases could lead to more negative memories, thoughts, and beliefs, thus explaining vital symptoms of anxiety such as nervousness and worrying. These results demonstrate that the altered positivity bias in anxious people extends into implicit low-level perceptual processing, which could be a crucial factor for successful intervention.

Anxiety is coupled with low self-esteem and negative self-view [8,11,12] which might relate to both the lack of self-prioritisation in anxious people and the emotional biases seen in more severe experiences of anxiety. The hypothesised changes in self-positivity were not directly supported by an interaction between self-prioritisation and emotional biases in the group-level analysis. We found that anxious people generally processed self-related input similar to other-related stimuli, which was different from the self-prioritisation effect in controls. The self is considered a robust construct which is immune to many biases—for example, the frequency-bias [44]—yet the self-prioritisation effect is eliminated in mental disorders such as anxiety and depression [45]. On the individual level, anxious people with severe anxiety perceived happy stimuli quicker and sad stimuli slower, but only for a shape that was related to others. This effect might also be interpreted as the self being robust and thus protected from the alteration in emotional biases, while the other-related stimuli is processed in a biased way in individuals with more severe anxiety. Another possible interpretation might be that anxious individuals perceive the other with greater esteem following the severity of anxiety [46]. In sum, there was a deficiency of self-prioritisation in anxiety coupled with altered emotional biases in more severe anxiety. One could assume that anxiety causes or is partly caused by a decreased self-prioritisation effect, but the change in perceptual positivity bias only occurs as the anxiety worsens. It certainly appears that the two biases interact differently and separately with anxiety.

One merit of the study is that it provides new support of an altered self-view in anxiety and suggests that using perceptual tasks to measure mental disorders may add helpful contributions to precision psychiatry. The self-prioritisation effect plays a crucial role in how we process information in the environment around us. According to the integrative theory of self, we use the self to bind important information from our environment together to form a comprehensive understanding of the world [47]. Humans have a bias towards self-related stimuli because these stimuli are crucial for understanding the world. However, as found in this study, people experiencing anxiety display altered self-prioritisation effects. Therefore, anxious individuals might have difficulty processing the information that is crucial for building a stable understanding of themselves and their environment. A lack of perceptual self-prioritisation is a feature of anxiety, which could explain the dysfunctional self-image and worldview characteristic of the disorder.

The current study raises several points of interest for further investigations. First, it is important to note that anxiety often co-occurs with depression and that people experiencing severe anxiety often also have depressive symptoms. Although this possible confound was controlled for in the current experiment, further work on the relations between the two factors is needed. Second, given that we used the sum of the GAD score rather than considering heterogeneity in individual cases, in-depth analysis with a large anxious sample would provide an informative way to assess individual variations of self-related deficits in anxiety. Traditional classifications of mental disorders focus heavily on symptoms. This form of classification is criticised for being too broad and for not always being helpful to the affected individual. With new measures and research on the underlying mechanisms of different disorders, it is becoming possible to individualise and personalise measurement tools and treatments [48]. The perceptual shape-label matching paradigm, or an adjusted version of it, could become an invaluable tool to clinicians and counsellors. If the task can reliably capture divergences in the self-prioritisation effect and positivity bias in anxiety, it can be used along with other clinical measures, providing a suitable and personalised treatment to the individual. Finally, the current study was explorative in nature and lacked a clear prediction from previous research. We found strong support of a diminished self-prioritisation effect and altered biases to valanced stimuli in sub-clinically anxious people. Future research should determine whether the same effects can be found in clinical anxiety. Being the first study of its kind to our knowledge, it will be useful to apply the self-emotional shape-label matching paradigm to future studies of anxiety with directional hypotheses.

## 5. Conclusions

This pilot study measured implicit perceptual biases to the self and emotional stimuli in anxious and non-anxious participants. The results show that the self-prioritisation effect was diminished in anxious individuals compared with healthy controls, and that an increase in other-related positivity bias predicted more severe anxiety in anxious individuals. The lack of self-prioritisation and the focus on positivity in others but not the self can be partially explained by the information-processing theory of anxiety. The findings highlight that implicit perceptual processes contribute to anxiety.

## Figures and Tables

**Table 1:** Overview of the trials in each of the participant groups and conditions.

		Match				Mismatch			
		Happy	Neutral	Sad	Line	Happy	Neutral	Sad	Line
Self-friend group ^1^	Self	20	20	20	20	20	20	20	20
	Friend *	20	20	20	20	20	20	20	20
Self-stranger group ^2^	Self	20	20	20	20	20	20	20	20
	Stranger *	20	20	20	20	20	20	20	20

Note: ^1^: N = 42, ^2^: N = 42, *: Data merged into one “other” category.

**Table 2:** Correlations between age, depression, anxiety, and reaction times to emotional self- and other-related stimuli in the anxious group.

Variables	M	SD	1	2	3	4	5	6	7	8
*Outcome variable*										
**1.** GAD	11.1	4.49	—							
*Control variable*										
**2.** Age	20.9	2.55	−0.14	—						
**3.** BDI ^1^	19.2	10.83	0.47 ***	−0.15						
*Predictor variables*					—					
**4.** Happy face, self-shape RTs	694.5	86.5	−0.10	0.07	−0.16	—				
**5.** Neutral face, self-shape RTs	706.7	99.46	−0.11	0.15	−0.11	0.73 ***	—			
**6.** Sad face, self-shape RTs	715.1	92.49	0.06	0.02	−0.02	0.72 ***	0.64 ***	—		
**7.** Happy face, other-shape RTs	723	109.91	−0.09	−0.04	−0.07	0.52 ***	0.55 ***	0.40 **	—	
**8.** Neutral face, other-shape RTs	720.2	105.13	0.02	−0.03	−0.13	0.51 ***	0.45 ***	0.41 ***	0.81 ***	
**9.** Sad face, other-shape RTs	740.4	108.64	0.18	−0.27 *	−0.00	0.47 ***	0.36 **	0.41 ***	0.75 ***	0.81 ***

Note. N = 60; M: mean; SD: standard deviation; * *p* < 0.05, ** *p* < 0.01, *** *p* < 0.001 (two-tailed tests). ^1^: Depression is a confounding variable which influences emotion perception [4]. It was measured with the Beck Depression Inventory (BDI-II) [42], which has 21 items with four statements each. Each statement has a rating of 0 (symptom not present) to 3 (severe symptom). Total scores < 9 = no depression, 10–18 = mild–moderate depression, 19–29 = moderate–severe depression, and > 30 = severe depression. The suicidal thoughts or wishes inventory was removed to avoid distress. The internal reliability was measured as high (Cronbach’s α = 0.925).

**Table 3:** Hierarchical multiple regression models for reaction time to facial expressions predicting GAD in the anxious group.

Predictors	R^2^	ΔR²	B	Beta	*t*
Step 1	0.23				
Age			−0.12	−0.05	−0.57
BDI			0.19	0.46	3.92 ***
Step 2	0.25	0.02			
Age			−0.08	−0.05	−0.40
BDI			0.18	0.44	3.64 ***
Happy face, self-shape RTs			−0.00	−0.07	−0.37
Neutral face, self-shape RTs			−0.01	−0.14	−0.79
Sad face, self-shape RTs			0.01	0.21	1.20
Step 3	0.36	0.11 *			
Age			0.10	0.06	0.45
BDI			0.18	0.44	3.71 ***
Happy face, self-shape RTs			−0.01	−0.15	−0.74
Neutral face, self-shape RTs			0.00	0.00	0.01
Sad face, self-shape RTs			0.01	0.12	0.70
Happy face, other-shape RTs			−0.02	−0.48	−2.24 *
Neutral face, other-shape RTs			0.00	0.08	0.34
Sad face, other-shape RTs			0.02	0.51	2.24 *

Note. N = 60; * *p* < 0.05, *** *p* < 0.001. B denotes unstandardized coefficients and Beta denotes standardized coefficients.

## Data Availability

The data used to support the findings of this study are available from the corresponding author upon request.

## Appendix A

**Table A1:** Friend- and stranger-related trials did not differ significantly on performance nor did they interact with emotional conditions.

Performance	Condition	F	df	*p*
Accuracy	All	0.30	4,79	0.878
	Happy	0.06	1,82	0.809
	Neutral	0.15	1,82	0.698
	Sad	0.24	1,82	0.628
	Line	0.72	1,82	0.399
Reaction Time	All	2.17	4,79	0.081
	Happy	1.25	1,82	0.266
	Neutral	0.01	1,82	0.925
	Sad	0.61	1,82	0.437
	Line	0.34	1,82	0.562

Note. MANCOVA analysis comparing “friend” and “stranger” responses. F = Wilks’ Lambda, df = degrees of freedom.

**Table A2:** Control condition (line) was similar to sad condition and significantly dissimilar to happy condition.

Performance	Condition		M	SD	Mean Difference	t	df	*p*
Accuracy (%)	Line		0.795	0.127				
		Happy	0.808	0.124	−0.013	−1.34	83	0.184
		Neutral	0.803	0.124	−0.008	−0.89	83	0.374
		Sad	0.796	0.114	<−0.001	−0.08	83	0.933
Reaction Time (ms)	Line		723.8	88				
		Happy	706.3	87.1	17.5	3.19	83	0.002 **
		Neutral	714.9	88	8.9	1.67	83	0.099
		Sad	723.2	81.8	0.6	0.1	83	0.919

Note. Paired samples *t*-test comparing the line condition to all other emotional conditions. M = mean, SD = standard deviation, t = Student’s statistic t between emotional and line conditions, df = degrees of freedom, ** *p* < 0.01.
